# Relationship between Football-Specific Training Characteristics and Tibial Bone Adaptation in Male Academy Football Players

**DOI:** 10.3390/sports11040086

**Published:** 2023-04-19

**Authors:** Ian Varley, Craig Sale, Julie P. Greeves, John G. Morris, Caroline Sunderland, Chris Saward

**Affiliations:** 1 Department of Sport Science, Sport, Health and Performance Enhancement (SHAPE) Research Centre, School of Science and Technology, Nottingham Trent University, Nottingham NG11 8NS, UK; 2 Department of Sport and Exercise Sciences, Institute of Sport, Manchester Metropolitan University, Manchester M1 7EL, UK; 3 Army Health and Performance Research, Army Headquarters, Andover SP11 8HJ, UK

**Keywords:** bone, exercise, soccer, loading, pQCT

## Abstract

We examined the relationship between football-specific training and changes in bone structural properties across a 12-week period in 15 male football players aged 16 years (Mean ± 1 SD = 16.6 ± 0.3 years) that belonged to a professional football academy. Tibial scans were performed at 4%, 14% and 38% sites using peripheral quantitative computed tomography immediately before and 12 weeks after increased football-specific training. Training was analysed using GPS to quantify peak speed, average speed, total distance and high-speed distance. Analyses were conducted with bias-corrected and accelerated bootstrapped 95% confidence intervals (BCa 95% CI). There were increases in bone mass at the 4% (mean ∆ = 0.15 g, BCa 95% CI = 0.07, 0.26 g, *g* = 0.72), 14% (mean ∆ = 0.04 g, BCa 95% CI = 0.02, 0.06 g, *g* = 1.20), and 38% sites (mean ∆ = 0.03 g, BCa 95% CI = 0.01, 0.05 g, *g* = 0.61). There were increases in trabecular density (4%), (mean ∆ = 3.57 mg·cm^−3^, BCa 95% CI = 0.38, 7.05 mg·cm^−3^, *g* = 0.53), cortical dentsity (14%) (mean ∆ = 5.08 mg·cm^−3^, BCa 95% CI = 0.19, 9.92 mg·cm^−3^, *g* = 0.49), and cortical density (38%) (mean ∆ = 6.32 mg·cm^−3^, BCa 95% CI = 4.31, 8.90 mg·cm^−3^, *g* = 1.22). Polar stress strain index (mean ∆ = 50.56 mm^3^, BCa 95% CI = 10.52, 109.95 mm^3^, *g* = 0.41), cortical area (mean ∆ = 2.12 mm^2^, BCa 95% CI = 0.09, 4.37 mm^2^, *g* = 0.48) and thickness (mean ∆ = 0.06 mm, BCa 95% CI = 0.01, 0.13 mm, *g* = 0.45) increased at the 38% site. Correlations revealed positive relationships between total distance and increased cortical density (38%) (r = 0.39, BCa 95% CI = 0.02, 0.66), and between peak speed and increased trabecular density (4%) (r = 0.43, BCa 95% CI = 0.03, 0.73). There were negative correlations between total (r = −0.21, BCa 95% CI = −0.65, −0.12) and high-speed distance (r = −0.29, BCa 95% CI = −0.57, −0.24) with increased polar stress strain index (38%). Results suggest that despite football training relating to increases in bone characteristics in male academy footballers, the specific training variables promoting adaptation over a 12-week period may vary. Further studies conducted over a longer period are required to fully elucidate the time-course of how certain football-specific training characteristics influence bone structural properties.

## 1. Introduction

Long-term, multi-directional weight-bearing exercise has a positive effect on bone accrual [1,2,3,4,5]. Football participation has been associated with increased bone mineral density (BMD) [6], bone size [7] and bone strength [8]. The high magnitude of loading that takes place during football training and match play [9], which leads to mechanotransduction [10], is thought to be the mechanism by which habitual football participation is associated with greater osteogenic effects compared with other sports in cross-sectional studies [5]. Surprisingly, the specific determinants of football participation, and physical activity in general, that cause an osteogenic response are not well established. 

Data from cross-sectional studies show that moderate- [11,12] and vigorous-intensity [13] habitual activity, measured via accelerometer-based activity monitors, are associated with increased bone strength [12,13]. Studies employing exercise interventions of various durations and intensities have shown positive bone adaptations in relation to the external loading experienced (i.e., the higher the assumed magnitude of external loading, the greater the bone response) [1,2]. The specific effects of magnitude of loading or how vigorous the movement is on bone accrual remain unclear and have been poorly defined. For example, the recently published position statement in the Journal of Bone Metabolism on increasing peak bone mass in adolescents recommends that 5–6 months of vigorous physical activity should be performed, but does not quantify the specific speed or intensity of activity required [14]. It is uncertain whether differing intensities of habitual exercise composed of equivalent mode and duration have the same or differing effects on the bone. It should be noted, however, that problems quantifying exercise intensity in a field setting and difficulties prescribing the same relative intensity for different individuals make the effects of exercise intensity a challenging area to investigate.

Bone health and strength during adolescence is pertinent since mechanical loading during these years is related to bone strength and fragility fracture prevention across the lifespan [15,16]. Habitual mechanical loading during pre-pubertal and pubertal stages of development has consistently been associated with current [17,18] and future [15] osteogenic effects. Furthermore, data from a large cohort of elite adolescent footballers show various increases in bone characteristics following a 12-week increase in training volume [7]. Knowledge about the type, intensity and duration of football-specific training related to bone structural changes would provide invaluable information for practitioners, which they could use to inform osteogenic training protocols.

Football training load has traditionally been expressed by the duration of training sessions and a coach’s subjective assessment of intensity (i.e., minutes of high-, medium- and low-intensity training). The validation of global positioning systems (GPS) in team sports [19] has allowed training load to be assessed in a more objective manner, involving the estimation of total distance covered and distance covered at certain speeds. Using GPS to quantify training may aid our understanding of the relationship between football-specific training and bone-related adaptations in young people. Therefore, the aim of the present study was to examine the relationship between football-specific training characteristics and changes in bone structural properties, during a 12-week period of increased training, in male academy footballers.

## 2. Materials and Methods

### 2.1. Participants

Participants were deemed eligible for the study if they were aged 16 years or above, injury-free, not currently taking any medication that influenced bone metabolism and had not received a joint replacement or prostheses. After reading the participant information sheet and having the opportunity to ask questions, participants signed a statement of informed consent, completed a pre-scan screening form (ensuring they met the inclusion criteria) and completed a health screen questionnaire. Forms were scrutinised by the lead investigator before the study commenced to confirm that participants met the inclusion/exclusion criteria. The study conformed to Ionising Radiation (Medical Exposure) Regulations and was approved by the National Health Service Research Ethics Committee (ethical application number:15/EM/0037).

#### 2.1.1. Professional Academy Youth Football Players

Fifteen first-year full-time male academy footballers aged 16.6 ± 0.3 years (Mean ± 1 SD) were recruited from a professional football academy through previously established relationships with Nottingham Trent University as part of the Bone Health in Elite Athlete Cohort (BEA-C). Academy players had a mass and stature of 74.4 ± 5.7 kg and 1.80 ± 0.08 m, respectively (Mean ± 1 SD). 

#### 2.1.2. Recreationally Active Youth Football Players

To avoid unnecessarily exposing individuals to the radiation associated with peripheral quantified computed tomography (pQCT) scans, control group data were derived from a previously published study conducted by the research group (see Varley et al. [7]). Thus, 13 males, recreationally active (played football habitually, as part of a non-elite club) football players aged 16.5 ± 0.5 years (Mean ± 1 SD) acted as the control group in the present study. These recreationally active football players had a mass and stature of 70.9 ± 4.0 kg and 1.75 ± 0.04 m, respectively (Mean ± 1 SD). 

### 2.2. Experimental Design

To determine whether there were changes in the study variables in the control group across the study period, the recreationally active footballers underwent assessments of stature, body mass and bone characteristics assessed using pQCT (XCT2000L, Stratec Medizintechnik, Pforzheim, Germany) once at baseline, and then again 12 weeks later. During this 12-week period, the control group maintained their normal physical activity (5.5 ± 4.8 h/wk). Paired-samples t-tests revealed that there were no changes (*p* > 0.05) in the stature, body mass or bone characteristics of the recreationally active footballers [7].

Professional academy footballers were tested during the first week of their pre-season training for the same baseline measurements as the control group. The academy footballers then completed 12 weeks of football-specific training and matches, which were monitored using GPS. After 12 weeks, the measurements conducted at baseline were repeated to establish changes in each pQCT variable for each academy footballer across the training period. In turn, for the academy footballers, this allowed changes in the pQCT variables to be correlated with GPS training load indicators to examine the relationships between training load and changes in bone characteristics. In addition, pQCT markers were normalised to body mass so that relationships between training load and changes in pQCT relative to body mass could be examined.

### 2.3. Football-Specific Training 

Academy footballers deemed of a suitable standard by their club graduate through the academy to become first year scholars. Although habitually accustomed to football training and match play as part of the academy at younger age groups, the start of the study was timed to coincide with their first experience of full-time training as a first-year scholar. This transition from part-time to full-time training was associated with a training volume increase of 62% (7.1 ± 1.2 to 11.5 ± 0.8 h·wk^−1^). All training was conducted and supervised by qualified coaches as part of their normal practice. Sessions consisted of gym sessions (e.g., mobilisation, fixed-cycle ergometer work, weight training), high-intensity running drills, small-sided games, technique-based drills, and matches. To assess physical demands in the 12-week period, players wore GPS units during outdoor training sessions. There were up to 71 outdoor sessions that players could participate in. Due to GPS unit availability, it was not always possible to monitor every player in every outdoor training session; yet on average, the number of outdoor sessions that players wore GPS was 40 ± 19 (Mean ± 1 SD). 

### 2.4. GPS Assessments of Football-Specific Training Characteristics

Football-specific training characteristics were assessed using a 10 Hz GPS system (Viper, STATSports, Newry, Ireland). This system has been validated for use by team sport players, demonstrating a bias of 1.80 ± 1.93% in peak speed during a 20 m sprint when assessed via GPS (26.3 ± 2.4 km.h^−1^) and radar gun (26.1 ± 2.6 km.h^−1^) [20]. Each player wore a harness containing a GPS unit positioned between the shoulder blades. Post-session, each GPS unit was downloaded to a laptop computer and analysed using commercially available software (Viper, STATSports, Ireland). The football-specific training characteristics of total distance covered (m), high-speed (>17.0 km.h^−1^) distance covered (m), average speed (km.h^−1^), and peak speed (km.h^−1^) were assessed for outdoor sessions using GPS. Variables were normalised to the total number and duration of sessions completed across the 12-week period.

### 2.5. pQCT Measurements

pQCT has been shown to provide a reliable measurement of tibial bone characteristics in humans [21]. pQCT scans of the dominant leg were performed by the same manufacturer-trained operator. The scanner was cross-calibrated prior to use, using manufacturer-supplied phantoms of a known density. The tibial length, determined as the midpoint of the medial malleolus to the medial aspect of the tibial plateau of each participant, was measured (nearest 1 mm). Before scanning commenced, the participant’s foot and leg were secured in the scanner using the manufacturer-supplied equipment and aligned using the integral laser. After the participant was secured, they were informed to refrain from any movement and a reference-point-locating scout-view scan was performed in the frontal plane to confirm the location of the middle of the distal end plate, which would serve as a reference point for the tibia. Scans of the distal sites (4%, 14%) and the diaphysis of the tibia (38%) from the positioning line took place. A voxel size set at 0.5 mm, a slice thickness of 2.5 mm, and a contour mode with a threshold of 180 mg·cm^−3^ were consistent for all measurements. A constant default threshold of 711 mg·cm^−3^ was used to assess trabecular bone and identify and remove cortical bone. The integral XCT2000L software (version 6.20A) (Stratec, Birkenfeld, Germany) was used to analyse the pQCT images.

If any movement artefacts (inaccuracies in the measurement caused by motion) were present following the scan, the image was classed as invalid, and a repeat measure was performed. If an artefact (visually identified by the experimenter following movement by the participant) was present in the second image, the participant was removed from the study, in line with the radiation exposure guidelines (no participants were removed from the current study on this basis). The following tibial measures were analysed: mass (4%, 14%, 38%, g), polar, Y and X stress strain index (14%, 38%, mm^3^), trabecular area (4%), trabecular density (4%, mg·cm^−3^), cortical area (14%, 38%), cortical density (14%, 38%, mg·cm^−3^) cortical thickness (14%, 38%, mm), periosteal circumference (14%, 38%, mm), endosteal circumference (14%, 38%, mm), and total area (14%, 38%, mm^2^). 

### 2.6. Data Analysis

Paired-samples *t*-tests were conducted to examine changes in bone characteristics before and after 12-weeks of football-specific training. Any significant changes in bone characteristics were followed up with Pearson correlations. That is, Pearson correlations were conducted to examine relationships between GPS variables and changes in pQCT variables in absolute (not relative to body mass) and relative (relative to body mass) terms. For the paired-samples *t*-tests, effect sizes were calculated using Hedges’s *g*, and for Pearson correlations, effect sizes were calculated using r-values [22]. Mean ± 1 SD were used to describe the average and variability of data, unless stated otherwise. Due to deviations from normality, data were analysed using robust bootstrapped procedures. Bootstrapping allows confidence intervals to be accurately estimated empirically for a given test statistic, and so all analyses were performed using bias-corrected and accelerated bootstrap confidence intervals with a 95% confidence level and 2000 resamples. Thus, instead of p-values, the present data were interpreted with regards to the bootstrapped confidence intervals and effect sizes, as this method has greater validity when distributions deviate from normality [23]. All analyses were conducted using IBM SPSS (v. 24) (IBM Corp, Armonk, NY, USA).

## 3. Results

On average (Mean ± 1 SD), across 12 weeks of football-specific training, academy players covered 313,621 ± 104,928 m of total distance and 17,667 ± 14,586 m of high-speed distance, and their peak and average speeds per session were 24.8 ± 2.5 km.h^−1^ and 5.5 ± 1.7 km.h^−1^. Table 1 shows the changes in bone characteristics across the 12-week period for the academy players. As previously reported, no changes in bone characteristics were shown in the control group [7].

Following 12 weeks of increased training, bone mass increased at the tibial 4% site (mean change = 0.15 g, BCa 95% CI = 0.07, 0.26 g, *g* = 0.72), 14% site (mean change = 0.04 g, BCa 95% CI = 0.02, 0.06 g, *g* = 1.20), and 38% site (mean change = 0.03 g, BCa 95% CI = 0.01, 0.05 g, *g* = 0.61). In terms of bone density, trabecular density at the 4% site increased (mean change = 3.57 mg·cm^−3^, BCa 95% CI = 0.38, 7.05 mg·cm^−3^, *g* = 0.53), cortical density at the 14% site increased (mean change = 5.08 mg·cm^−3^, BCa 95% CI = 0.19, 9.92 mg·cm^−3^, *g* = 0.49), and cortical density at the 38% site increased (mean change = 6.32 mg·cm^−3^, BCa 95% CI = 4.31, 8.90 mg·cm^−3^, *g* = 1.22). Furthermore, polar stress strain index (mean change = 50.56 mm^3^, BCa 95% CI = 10.52, 109.95 mm^3^, *g* = 0.41), cortical area (mean change = 2.12 mm^2^, BCa 95% CI = 0.09, 4.37 mm^2^, *g* = 0.48) and cortical thickness (mean change = 0.06 mm, BCa 95% CI = 0.01, 0.13 mm, *g* = 0.45) increased at the 38% site. There were no changes in other bone characteristics (see Table 1).

**Figure 1:**
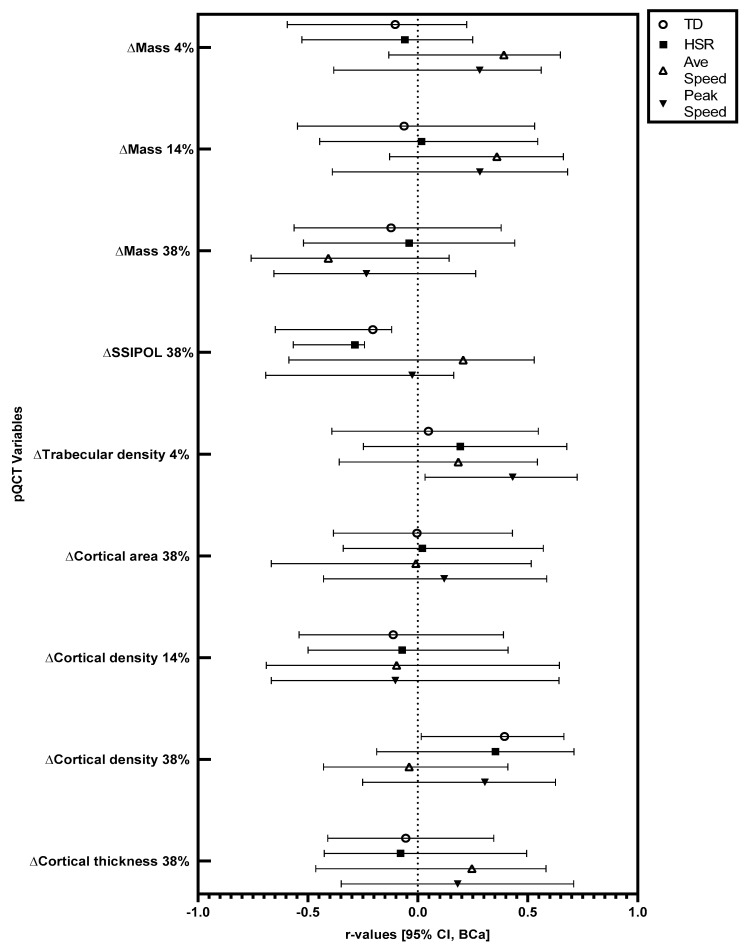
Relationships between training load variables and changes in bone characteristics across 12 weeks of football-specific training. r-values and associated error bars are displayed. Error bars are bias-corrected and accelerated bootstrap confidence intervals with a 95% confidence level. TD: total distance, HSR: high-speed running distance, Ave Speed: average speed, SSI: stress strain index, POL: polar.

**Figure 2:**
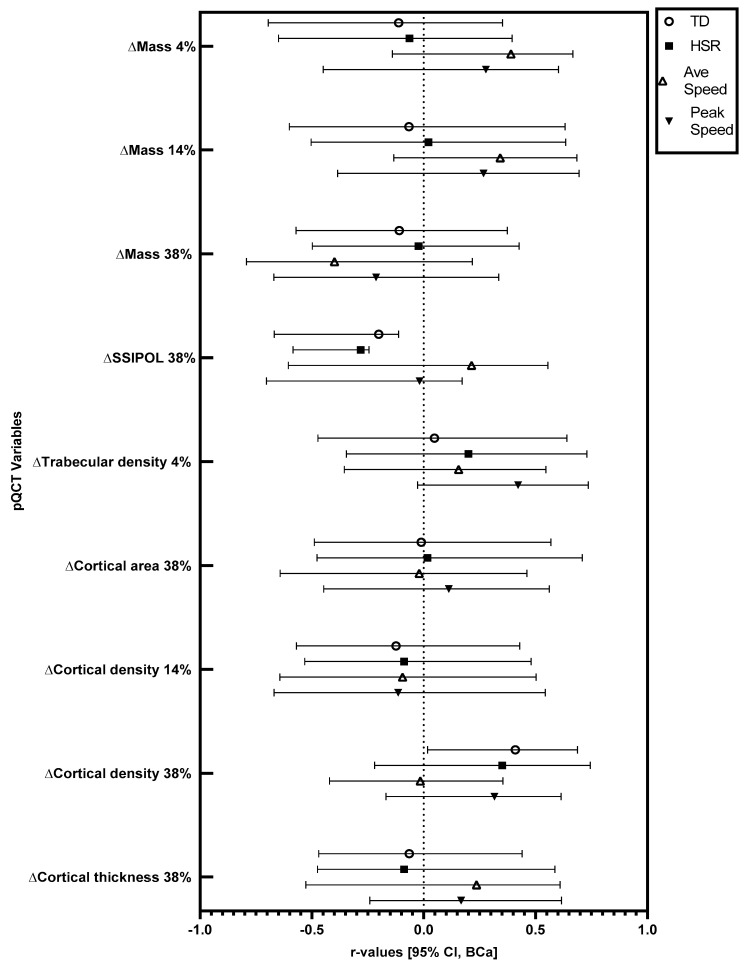
Relationships between training load variables and changes in bone characteristics relative to body mass, across 12 weeks of football-specific training. r-values and associated error bars are displayed. Error bars are bias-corrected and accelerated bootstrap confidence intervals with a 95% confidence level. TD: total distance, HSR: high-speed running distance, Ave Speed: average speed, SSI: stress strain index, POL: polar.

Significant changes in pQCT variables were followed up with correlations. Relationships at the 95% CI (BCa) level between training load variables and changes in pQCT variables in absolute (not relative to body mass) and relative (relative to body mass) terms are displayed in Figure 1 and Figure 2. 

In absolute terms, there were significant positive correlations between total running distance and the observed increase (mean increase = 6.32 mg·cm^−3^) in cortical density at the 38% site (r = 0.39, BCa 95% CI = 0.02, 0.66), and between peak speed and the observed increase (mean increase = 3.57 mg·cm^−3^) in trabecular density at the 4% site (r = 0.43, BCa 95% CI = 0.03, 0.73). In absolute terms, there were significant negative correlations between total running distance (r = −0.21, BCa 95% CI = −0.65, −0.12) and high-speed running distance (r = −0.29, BCa 95% CI = −0.57, −0.24) with the observed increase (mean increase = 50.56 mm^3^) in polar stress strain index at the 38% site. When considered relative to body mass, there was a significant positive correlation between total running distance and the observed increase in cortical density at the 38% site (r = 0.41, BCa 95% CI = 0.02, 0.69). Relative to body mass, there were significant negative correlations between total running distance (r = −0.20, BCa 95% CI = −0.67, −0.11) and high-speed running distance (r = −0.28, BCa 95% CI = −0.58, −0.24) with the observed increase in polar stress strain index at the 38% site.

## 4. Discussion

The present study has shown that 12 weeks of increased football-specific training is related to an increase in numerous tibial bone characteristics in academy footballers (Table 1). For the first time, it has been shown that peak speed is positively related to increases in trabecular density (4%), while distance covered is positively related to increases in cortical density (38%) in academy footballers following 12 weeks of increased training. Tibial polar bone strength (38% site) increased across the 12-week period; however, this increase was negatively related to total distance and distance covered at high speed. As bone strength is an important factor in skeletal injury avoidance across a lifespan, these findings may have implications for those undertaking and/or prescribing exercise inventions to optimise bone health. 

The present study shows that increased training over a 12-week period relates to increases in a range of bone characteristics in professional academy footballers. Conversely, the control group maintained their training over a 12-week period and no changes in bone characteristics were observed. The bone characteristics shown to increase are different from those reported by Varley et al. [7]. The reason for these differences could be due to the participants in the present study being recruited from only one academy, while the participants in the Varley et al. [7] were from five academies. The difference in the specific training styles and intensities may have caused different bone adaptations. No changes in the bone characteristics of the control were expected; due to the lack of change in training volume, it is unlikely that enough stimulus occurred to induce an increase in mechanotransduction [10]. Habitual football participation has been associated with a greater bone mass in comparison to resistance training [24], swimming [25] and sedentary lifestyles [2]. However, as the specific intensity and volume of football activity that causes osteogenic effects is not known, therefore, the use or prescription of football training as a method of optimising bone health is limited. The findings from the present study may be a useful addition for sports practitioners and health professionals who are looking to create osteogenic exercise interventions.

A greater peak running speed during 12 weeks of football-specific training was positively related to increased trabecular density (4% tibial site) in participants already accustomed to football-specific training. Running at high speed has previously been shown to elicit a greater ground reaction force than slower speeds and, therefore, a greater assumed load [26]. The greater magnitude of force produced by faster running speeds is likely to have resulted in a greater mechanotransductive response and resulted in an increase in bone remodelling and subsequently trabecular density. In line with these findings, it has been shown that running for 60 min at 75% VO_2max_ resulted in an increased bone resorption (β-CTX) following exercise, although no such changes were shown at 55% and 65% VO_2max_. The higher-intensity trial may have caused greater mechanical loading, which may be the reason for the association with increased osteogenesis [27]. In the present study, the relationship between increased trabecular density and speed of movement suggests that the magnitude of the loading is an important factor in bone adaptation. Rodent models agree with this assertion, having shown that short volumes (72 s/day) of high mechanical load are enough to cause positive bone adaptations [28]. Similarly, physical activity incorporating a jumping component has been shown to have a greater positive effect on tibial cross-sectional area [29], femoral neck BMD [30] and spine and hip bone mineral content (BMC) [31] when compared to weight-bearing activities that do not involve jumping. 

Total distance covered was positively related to increases in cortical density at the 38% tibial site. This correlation may be due to the likelihood that a greater distance covered could have resulted in a greater number of osteogenic actions, such as accelerations, decelerations, and changes in direction (Varley et al. [32]), due to these actions being necessitated by football training and match play. Specifically, decelerations result in greater force production in comparison to continuous activities [33], which is likely to cause an increased bone load. Whole-body BMD is greater in football players compared to long-distance runners [34] although long-distance running does not typically involve multiple changes in direction, which may explain why long-distance running and total distance covered during football training have seemingly contrasting findings. 

Despite the polar stress strain index increasing during the 12 weeks of increased training overall, negative relationships were shown between increases in polar stress strain index (38% tibial site) and total distance and distance at high speed. These negative correlations may possibly reflect an initial increase in bone resorption caused by training [35], which may induce an increase in bone formation and subsequent bone accrual following the study period. The contrasting correlations between distance covered with cortical density changes and polar stress strain index changes may be a result of differences in inter-individual response to the increased volume of football training. Nevertheless, given that BMD is a factor in the determination of stress strain index, it would be expected that both metrics would change in the same direction. The reason for changes not being universally shown at all measured skeletal sites may be related to a variety of factors. Bone responds to loading in a site-specific manner and therefore bone may have increased in size and shape in one direction (e.g., anteriorly) but decreased in another (e.g., posteriorly), causing no witnessed net change in the bone parameter despite changes taking place [36]. It is advised that segmental analysis of tibial structures is conducted in future studies to test this hypothesis. Stress fracture injuries commonly occur in the vicinity of the 38% site of the tibia [37]. This could be a result of the greater bending moments that occur at the 38% site and may explain why an increase in bone strength at the 38% site of the tibia was shown but not at the 14% and 4% sites. The time course of how individual bone characteristics respond to certain exercise demands is complex, with different exercise intensities and types eliciting varying bone responses [35].

Furthermore, several bone characteristics did not change across the study period (see Table 1). In addition, for several of the bone characteristics that did change, there was no relationship between these observed changes and GPS-derived training load variables (see Figure 1 and Figure 2). This could have been due to the relatively short duration of the training period. Indeed, the median bone remodelling cycle in cortical bone is 120 days [38], with the current training period being around 84 days and involving 83 ± 16 sessions, meaning that potential changes might not have been completed in the training period. Additionally, as the players were accustomed to the type of exercise being conducted, the expected adaptations in bone are likely to be smaller and/or take longer to manifest compared to individuals unaccustomed to a type of exercise. Nevertheless, that we showed several relationships between bone changes and training characteristics, despite the participants being accustomed to the activity, and the training period only being 12 weeks, suggests that football training may be a particularly relevant mode of exercise for eliciting bone changes. It should, however, be noted that unmeasured training load factors not captured by the GPS system, such as strength and conditioning-related training, may have influenced the findings. Further investigations are required that assess the time-course of bone adaptation and specific bone component responses to exercise. 

Population-based studies assessing exercise and bone adaptation have shown that vigorous physical activity (VPA) is significantly associated with tibial strength in a large cohort of adolescent girls and boys [12] and young adults [13]. In addition, moderate to vigorous physical activity (MVPA) and VPA were independent predictors of tibial shaft strength in pre-pubertal children [11]. Unlike the current study, the mode of exercise undertaken by the participants in these studies was not recorded. Therefore, the suggestion that bone adaptation is taking place because of the mechanotransductive response to weight-bearing exercise cannot be confirmed. As the current study used a known mode of exercise (football-specific training), the mechanism of adaptation can be more clearly evidenced. Thus, it would appear that, based on the results of the current 12-week study, even relatively short periods of high-speed physical activity positively relate to bone accrual, even in individuals with a history of structured physical activity. 

The present study is not without limitations, including the fact that the GPS unit was mounted on the upper back and discrepancies between movement at the tibia and back could have occurred. Given the nature of the activity, however, it was not practical to position the GPS on the tibia, and previous studies [19,20] that have validated the use of GPS for activity tracking have mounted the unit on the upper back. The use of GPS offers a validated, practical method of capturing performance-related variables, offering a greater degree of accuracy when compared to accelerometery. Moreover, indoor gym sessions were undertaken by the academy footballers, but this type of activity was not captured by the GPS system used in the present study. Lastly, unmeasured factors, such as genetics, maturation status, longitudinal external load and diet may also have contributed to the differences in bone characteristics. However, assessing these factors in a population such as elite football players is extremely challenging due to both practical and logistical issues. 

Future research should look to further quantify mechanical loading and bone adaptations in elite populations by measuring mechanical load with a greater degree of precision over a longer period to further elucidate the effects of exercise on bone characteristics. Furthermore, future studies are recommended that assess the interaction between activity variables (e.g., the interaction between the duration and volume of high-speed and high-distance activity compared to high-speed and low-distance) and bone characteristics to fully clarify the relationship between the high-magnitude loading and optimised bone accrual. The assessment of acceleration and deceleration variables is also recommended, as these aspects of exercise typically involve elements of high-magnitude loading. 

## 5. Conclusions

The aim of the present study was to examine the relationship between football-specific training characteristics and changes in bone structural properties during a 12-week period of increased training in male academy footballers. The results of the present study indicated that 12 weeks of increased training was positively related to tibial bone adaptation in male academy footballers. Football-specific training characteristics were related to changes in bone structural properties, whereby peak speed was positively related to increases in trabecular density (4%), distance covered was positively related to increases in cortical density (38%), and total distance and distance covered at high speed were negatively related to increases in tibial polar bone strength (38% site). These results suggest that despite football training relating to increases in bone characteristics in male academy footballers, the specific training variables promoting adaptation over a 12-week period may vary. Further studies conducted over a longer period of time that measure multiple determinants of bone adaption are required to fully elucidate the time-course of how certain football-specific training characteristics influence bone structural properties.

## Figures and Tables

**Table 1:** Changes in bone characteristics following 12 weeks of football-specific training.

Bone Characteristics	Pre (M. ± SD)	Post (M. ± SD)	Mean Change	95% CI of Change	Effect Size (*g*)
Bone mass 4% (g)	4.79 ± 0.44	4.94 ± 0.41	0.15	[0.07, 0.26] *	0.72
Bone mass 14% (g)	3.35 ± 0.31	3.39 ± 0.31	0.04	[0.02, 0.06] *	1.20
Bone mass 38% (g)	4.54 ± 0.38	4.57 ± 0.37	0.03	[0.01, 0.05] *	0.61
Polar stress strain index 14% (mm^3^)	2217 ± 380	2234 ± 373	17.38	[−78.63, 81.10]	0.10
Polar stress strain index 38% (mm^3^)	2298 ± 341	2348 ± 316	50.56	[10.52, 109.95] *	0.41
X stress strain index 14% (mm^3^)	1230 ± 179	1242 ± 174	12.16	[−23.64, 44.11]	0.20
X stress strain index 38% (mm^3^)	1467 ± 247	1492 ± 230	25.00	[−2.28, 58.63]	0.36
Y stress strain index 14% (mm^3^)	1290 ± 160	1310 ± 150	19.31	[−11.19, 49.12]	0.37
Y stress strain index 38% (mm^3^)	1309 ± 167	1310 ± 186	0.90	[−23.77, 22.27]	0.02
Trabecular area 4%, (mm^2^)	615 ± 59	627 ± 57	11.97	[−2.01, 34.56]	0.27
Trabecular density 4% (mg·cm^−3^)	286 ± 35	289 ± 34	3.57	[0.38, 7.05] *	0.53
Cortical area 14% (mm^2^)	223 ± 19	224 ± 19	1.28	[−1.45, 3.82]	0.24
Cortical area 38% (mm^2^)	378 ± 33	380 ± 31	2.12	[0.09, 4.37] *	0.48
Cortical density 14% (mg·cm^−3^)	1064 ± 31	1070 ± 26	5.08	[0.19, 9.92] *	0.49
Cortical density 38% (mg·cm^−3^)	1117 ± 25	1123 ± 25	6.32	[4.31, 8.90] *	1.22
Cortical thickness 14% (mm)	2.87 ± 0.33	2.87 ± 0.29	0.00	[−0.06, 0.05]	0.01
Cortical thickness 38% (mm)	6.12 ± 0.56	6.18 ± 0.51	0.06	[0.01, 0.13] *	0.45
Periosteal circumference 14% (mm)	86.9 ± 4.5	87.3 ± 4.6	0.37	[−0.06, 0.92]	0.34
Periosteal circumference 38% (mm)	81.2 ± 3.9	81.1 ± 3.8	−0.13	[−0.57, 0.16]	−0.18
Endosteal circumference 14% (mm)	68.9 ± 5.8	69.2 ± 5.7	0.36	[−0.38, 1.34]	0.20
Endosteal circumference 38% (mm)	42.8 ± 5.4	42.3 ± 5.0	−0.52	[−1.44, 0.01]	−0.34

Note: Pre- and post-training data are presented as means and standard deviations. A 95% CI of change = bias-corrected and accelerated bootstrapped 95% confidence intervals for the mean changes between pre- and post-training. * signifies that mean change estimates do not cross zero at the 95% confidence level. The size of effects pre- and post-training are represented using Hedges’s *g.*

## Data Availability

Due to the nature of this research, participants of this study did not agree for their data to be shared publicly, so supporting data are not available.

## References

[1] Greene D.A., Naughton G.A., Bradshaw E., Moresi M., Ducher G. (2012). Mechanical loading with or without weight-bearing activity: Influence on bone strength index in elite female adolescent athletes engaged in water polo, gymnastics, and track-and-field. J. Bone Miner. Metab..

[2] Nilsson M., Ohlsson C., Odén A., Mellström D., Lorentzon M. (2012). Increased physical activity is associated with enhanced development of peak bone mass in men: A five-year longitudinal study. J. Bone Miner. Res..

[3] Weidauer L., Eilers M., Binkley T., Vukovich M., Specker B. (2012). Effect of different collegiate sports on cortical bone in the tibia. J. Musculoskelet. Neuronal Interact..

[4] Wilks D.C., Winwood K., Gilliver S., Kwiet A., Chatfield M., Michaelis I., Sun L., Ferretti J.L., Sargeant A.J., Felsenberg D. (2009). Bone mass and geometry of the tibia and the radius of master sprinters, middle and long distance runners, race-walkers and sedentary control participants: A pQCT study. Bone.

[5] Creighton D.L., Morgan A.L., Boardley D., Brolinson P.G. (2001). Weight-bearing exercise and markers of bone turnover in female athletes. J. Appl. Physiol..

[6] Krustrup P., Helge E.W., Hansen P.R., Aagaard P., Hagman M., Randers M.B., de Sousa M., Mohr M. (2018). Effects of recreational football on women’s fitness and health: Adaptations and mechanisms. Eur. J. Appl. Physiol..

[7] Varley I., Hughes D.C., Greeves J.P., Fraser W.D., Sale C. (2017). Increased training volume improves bone density and cortical area in adolescent football players. Int. J. Sport. Med..

[8] Vlachopoulos D., Ubago-Guisado E., Barker A.R., Metcalf B.S., Fatouros I.G., Avloniti A., Knapp K.M., Moreno L.A., Williams C.A., Gracia-Marco L. (2017). Determinants of bone outcomes in adolescent athletes at baseline: The PRO-BONE study. Med. Sci. Sport. Exerc..

[9] Harry J.R., Barker L.A., Mercer J.A., Dufek J.S. (2017). Vertical and horizontal impact force comparison during jump landings with and without rotation in NCAA Division I male soccer players. J. Strength Cond. Res..

[10] Bonewald L.F. (2007). Osteocytes as dynamic multifunctional cells. Ann. N. Y. Acad. Sci..

[11] Kehrig A.M., Björkman K.M., Muhajarine N., Johnston J.D., Kontulainen S.A. (2019). Moderate to vigorous physical activity and impact loading independently predict variance in bone strength at the tibia but not at the radius in children. Appl. Physiol. Nutr. Metab..

[12] Tobias J.H., Steer C.D., Mattocks C.G., Riddoch C., Ness A.R. (2007). Habitual levels of physical activity influence bone mass in 11-year-old children from the United Kingdom: Findings from a large population-based cohort. J. Bone Miner. Res..

[13] Gabel L., Macdonald H.M., Nettlefold L., McKay H.A. (2017). Physical activity, sedentary time, and bone strength from childhood to early adulthood: A mixed longitudinal HR-pQCT study. J. Bone Miner. Res..

[14] Min S.K., Oh T., Kim S.H., Cho J., Chung H.Y., Park D.H., Kim C.S. (2019). Position Statement: Exercise guidelines to increase peak bone mass in adolescents. J. Bone Metab..

[15] Nilsson M., Ohlsson C., Mellström D., Lorentzon M. (2009). Previous sport activity during childhood and adolescence is associated with increased cortical bone size in young adult men. J. Bone Miner. Res..

[16] Tenforde A.S., Fredericson M. (2011). Influence of sports participation on bone health in the young athlete: A review of the literature. PM&R.

[17] Lorentzon M., Mellström D., Ohlsson C. (2005). Association of amount of physical activity with cortical bone size and trabecular volumetric BMD in young adult men: The GOOD study. J. Bone Miner. Res..

[18] Specker B., Binkley T.L. (2004). Increased periosteal circumference remains present 12 months after an exercise intervention in preschool children. Bone.

[19] MacLeod H., Morris J., Nevill A., Sunderland C. (2009). The validity of a non-differential global positioning system for assessing player movement patterns in field hockey. J. Sport. Sci..

[20] Beato M., Devereux G., Stiff A. (2018). Validity and reliability of global positioning system units (STATSports Viper) for measuring distance and peak speed in sports. J. Strength Cond. Res..

[21] Ashe M.C., Khan K.M., Kontulainen S.A., Guy P., Liu D., Beck T.J., McKay H.A. (2006). Accuracy of pQCT for evaluating the aged human radius: An ashing, histomorphometry and failure load investigation. Osteoporos. Int..

[22] Cohen J. (1988). Statistical Power Analysis for the Behavioral Sciences.

[23] Field A.P., Wilcox R.R. (2017). Robust statistical methods: A primer for clinical psychology and experimental psychopathology researchers. Behav. Res. Ther..

[24] Helge E.W., Aagaard P., Jakobsen M.D., Sundstrup E., Randers M.B., Karlsson M.K., Krustrup P. (2010). Recreational football training decreases risk factors for bone fractures in untrained premenopausal women. Scand. J. Med. Sci. Sport..

[25] Nikander R., Sievänen H., Uusi-Rasi K., Heinonen A., Kannus P. (2006). Loading modalities and bone structures at nonweight-bearing upper extremity and weight-bearing lower extremity: A pQCT study of adult female athletes. Bone.

[26] Weyand P.G., Sternlight D.B., Bellizzi M.J., Wright S. (2001). Faster top running speeds are achieved with greater ground forces not more rapid leg movements. J. Appl. Physiol..

[27] Lin C., Huang T., Tu K., Lin L.L., Tu Y., Yang R. (2012). Acute effects of plyometric jumping and intermittent running on serum bone markers in young males. Eur. J. Appl. Physiol..

[28] Rubin C.T., Lanyon L.E. (1984). Regulation of bone formation by applied dynamic loads. J. Bone Jt. Surg. Am..

[29] Nilsson M., Ohlsson C., Sundh D., Mellström D., Lorentzon M. (2010). Association of physical activity with trabecular microstructure and cortical bone at distal tibia and radius in young adult men. J. Clin. Endocrinol. Metab..

[30] Allison S.J., Poole K.E., Treece G.M., Gee A.H., Tonkin C., Rennie W.J., Folland J.P., Summers G.D., Brooke-Wavell K. (2015). The influence of high-impact exercise on cortical and trabecular bone mineral content and 3d distribution across the proximal femur in older men: A randomized controlled unilateral intervention. J. Bone Miner. Res..

[31] Gunter K.B., Almstedt H.C., Janz K.F. (2012). Physical activity in childhood may be the key to optimizing lifespan skeletal health. Exerc. Sport. Sci. Rev..

[32] Varley I., Ward M., Thorpe C., Beardsley N., Greeves J., Sale C., Saward C. (2023). External training load is associated with adaptation in bone and body composition over the course of a season in elite male footballers. Bone Rep..

[33] Dalen T., Jørgen I., Gertjan E., Havard G., Ulrik W. (2016). Player load, acceleration, and deceleration during forty-five competitive matches of elite soccer. J. Strength Cond. Res..

[34] Fredericson M., Chew K., Ngo J., Cleek T., Kiratli J., Cobb K. (2007). Regional bone mineral density in male athletes: A comparison of soccer players, runners and controls. Br. J. Sport. Med..

[35] Dolan E., Varley I., Ackerman K.E., Pereira R.M.R., Elliott-Sale K.J., Sale C. (2020). The bone metabolic response to exercise and nutrition. Exerc. Sport. Sci. Rev..

[36] Weatherholt A.M., Warden S.J. (2016). Tibial bone strength is enhanced in the jump leg of collegiate-level jumping athletes: A within-subject controlled cross-sectional study. Calcif. Tissue Int..

[37] Wall J., Feller J.F. (2006). Imaging of stress fractures in runners. Clin. Sport. Med..

[38] Agerbaek M.O., Eriksen E.F., Kragstrup J., Mosekilde L., Melsen F. (1991). A reconstruction of the remodelling cycle in normal human cortical iliac bone. Bone Miner..

